# Paracetamol-induced skin blood flow and blood pressure changes

**DOI:** 10.1186/cc9765

**Published:** 2011-03-11

**Authors:** M Boyle, A Lau, L Nicholson, M O'Brien, G Flynn, D Collins, W Walsh, D Bihari

**Affiliations:** 1 Prince of Wales Hospital, Randwick, NSW, Australia; 2 University of NSW, Randwick, NSW, Australia

## Introduction

Paracetamol given for fever is associated with hypotension [1]. Spectral analyses (Fourier, wavelet) can be used to identify low-frequency oscillations of skin blood flow (skBF) [2]. The relationship of paracetamol to skBF and blood pressure (BP) in febrile patients was studied.

## Methods

Twenty-nine adults, 58 ± 15 years, were treated with enteral or intravenous paracetamol for fever. Forty-one percent (*n *= 12) were medical, 31% (*n *= 9) surgical, and 28% (*n *= 8) neurological. APACHE II score was 17.2 ± 8.3. Frequency domain analyses of the laser Doppler flowmetry (LDF) waveforms of two patients were undertaken. Both patients (A and B) had good LDF waveforms, both increased skBF whilst BP fell in patient B.

## Results

**Figure 1:**
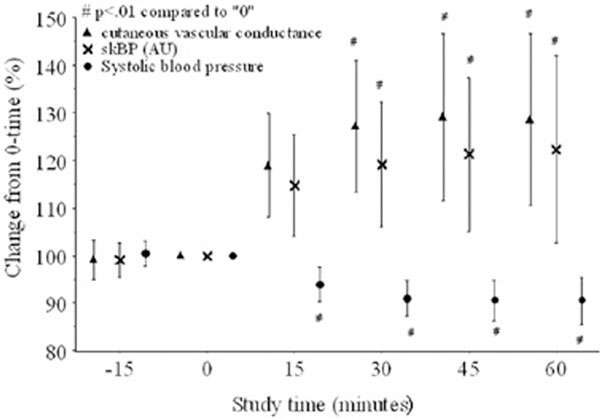


Temperature, BP and skBF were recorded 15 minutes prior to paracetamol, at administration (T0) and then every 15 minutes for 60 minutes. Thirty datasets were recorded. Temperature at T0 was 38.7 ± 0.6°C. BP decreased over the study period whilst skBF and cutaneous vascular conductance (CVC = skBF/mean arterial pressure) increased (repeated-measures ANOVA, *P *< 0.05). Systolic BP decreased (*P *< 0.01) at all post-administration times and was 90 ± 13% of T0 at 60 minutes (Figure 1). CVC was 128 ± 48% of T0 at 60 minutes. Systolic BP fell significantly (≥15%) in 17 patients (59%). Normalised average power spectral density (PSD) increased substantially in the 0.40 to 2.0 Hz band in patient A, corresponding to an increase in cardiac output (CO). Wavelet scalograms showed increased relative energy for < 0.012 Hz (patients A and B) consistent with cutaneous vasodilation and around 0.02 Hz (patient A) consistent with increased sympathetic activity [2].

## Conclusions

Paracetamol induced increases in skBF consistent with its antipyretic action. Changes in PSD and wavelet analysis were consistent with cutaneous vasodilation.
